# Functional human skin explants as tools for assessing mast cell activation and inhibition

**DOI:** 10.3389/falgy.2024.1373511

**Published:** 2024-03-27

**Authors:** Clarence Rachel Villanueva, Keane Barksdale, Tinuola Owolabi, Donavan Bridges, Kristin Chichester, Sarbjit Saini, Eric T. Oliver

**Affiliations:** Division of Allergy and Clinical Immunology, Department of Medicine, Johns Hopkins University School of Medicine, Baltimore, MD, United States

**Keywords:** mast cell, histamine, explant, IgE, BTK, MRGPRX2

## Abstract

Mast cells are activated through a variety of different receptors to release preformed granules and mediators synthesized *de novo*. However, the physiology and function of mast cells are not fully understood. Traditional studies of mast cell activation in humans have utilized cultures of tissue-derived mast cells including CD34+ progenitor cells or well-characterized commercially available cell lines. One limitation of these methods is that mast cells are no longer in a natural state. Therefore, their applicability to human skin disorders may be limited. Human skin explant models have been utilized to investigate the short-term effects of cell mediators, drugs, and irritants on skin while avoiding the ethical concerns surrounding *in vivo* stimulation studies with non-approved agents. Nonetheless, few studies have utilized intact human tissue to study mast cell degranulation. This “Methods” paper describes the development and application of an intact skin explant model to study human mast cell activation. In this manuscript, we share our protocol for setting up *ex vivo* human skin explants and describe the results of stimulation experiments and techniques to minimize trauma-induced histamine release. Skin explants were generated using de-identified, full-thickness, non-diseased skin specimens from plastic and reconstructive surgeries. Results were reproducible and demonstrated FcɛRI- and MRGPRX2-induced mediator release which was inhibited with the use of a BTK inhibitor and QWF, respectively. Thus, this explant model provides a quick and accessible method of assessing human skin mast cell activation and inhibition.

## Introduction

Mast cell activation is implicated in a diverse range of allergic disorders and innate immune processes (1). FcɛRI-IgE binding is perhaps one of the most well-known pathways for mast cell activation. FcɛRI is a high-affinity heterotetramer receptor for IgE containing one α-, one β-, and a γ-chain homodimer (αβγ2). The α-chain subunit facilitates the IgE binding. Meanwhile, the β-chain and γ-chain subunits allow for the downstream signaling cascade via Lyn inducing transphosphorylation of immunoreceptor tyrosine-based activation motifs (ITAMs) that are attached to both chain subunits (2). In addition to Lyn, the Fc*ε*RI pathway features multiple downstream kinases including Syk, BTK, Fyn, and PI3K (3). Notably, inhibition of BTK, Syk, or PI3K broadly prevents IgE-mediated degranulation and cytokine production in primary human mast cells (3–7). The function of BTK in mast cells was further delineated in a study by Kawakami et al. (8). The BTK inhibitor acalabrutinib was shown to prevent anaphylaxis completely in a humanized mouse model and in most peanut allergic individuals during an oral food challenge (3, 9).

A more recently discovered pathway for activating mast cell degranulation is via Mas-related G protein-coupled receptor member X2 (MRGPRX2) (10). Like FcɛRI, MRGPRX2 signals through pertussis toxin (PTX)-sensitive G_i_ proteins that control exocytosis (11, 12). MRGPRX2 is expressed at high levels in human skin mast cells and synovial mast cells, but not in lung mast cells (13). However, unlike FcɛRI, MRGPRX2 is a low-affinity, “universal” receptor for both endogenous and exogenous ligands with shared properties of cationic molecules and amphipathic peptides (10, 14). In addition, McNeil et al. demonstrated that high concentrations of MRGPRX2 ligands induced swelling and anaphylaxis in mice via the murine orthologue Mrgprb2 (15).

Through these and other pathways, mast cells are activated to rapidly release preformed granules containing proteases, biogenic amines, and cytokines- all of which are vital in inflammatory responses (7, 16, 17). Such preformed granules include tryptase, histamine, and Tumor necrosis alpha (TNF-α). Tryptase activates protease activated receptors (PARs) on a variety of cell types including sensory neurons. Histamine enables the clinical features of allergic reactions. TNF-α stimulates the expression of adhesion molecules, which attract and bind leukocytes to the inflamed site (7, 17, 18). Synthesized mediators such as leukotrienes and prostaglandin D_2_ can potentiate the vasodilatory effects of histamine and serve to recruit and activate leukocytes. Therefore, activation of mast cells can lead to degranulation and generation of mediators implicated in angioedema, atopic dermatitis, anaphylaxis, asthma, rosacea, and chronic urticaria (19). Thus, inhibition of mast cell activation is a viable target for treatment (7, 20–22).

Traditional studies of mast cell activation in humans have utilized cultures of mast cells derived from tissue or CD34+ progenitor cells, or well-characterized commercially available cell lines.^12^ Umbilical cord blood, peripheral blood mononuclear cells (PBMCs), or bone marrow mononuclear cells (BMMCs) have been essential for procuring CD34+ progenitor cells to generate human mast cells (23). However, the process of generating mast cell cultures from skin homogenates or CD34+ progenitors can be costly and take 4 weeks and 6 weeks, respectively (24). An advantage of utilizing commercially available cell lines is that they can be utilized immediately for the study of immunological responses via mast cell stimulation. Available mast cell lines include HuMCs (human mast cells cultured from CD34+ progenitor cells), LAD2 (Laboratory of allergic diseases 2) mast cells, and the HMC1 (Human Mast Cell 1) line. Compared to human skin mast cells, both LAD2 and HMC1 mast cells have significantly lower levels of tryptase and chymase (25). HuMCs and LAD2 cells both have a slow growth rate thus limiting their expansion (26). A universal limitation of all of these methods is that mast cells are no longer in a natural state. Therefore, their applicability to human skin disorders may be limited.

An alternative method for assessing mast cell activation is microdialysis. Skin microdialysis is a technique used to recover soluble endogenous and exogenous molecules from the interstitial space in human skin (27). This type of procedure can be performed *in vivo* or *ex vivo*. It involves the insertion of thin tubular dialysis membranes into the dermis or the subcutaneous tissue which are then perfused at a low speed with a physiological solution. Due to its invasive nature, a local anesthetic is generally required to ease discomfort from probe insertion (27). Soluble molecules present in the extracellular fluid diffuse into the microdialysis tube which are then collected for analysis. Appropriate controls are required to determine whether sampled molecules are truly related to the disease state under investigation or have been generated as part of the skin response to probe implantation (27). Microdialysis has been utilized to assess mast cell MRGPRX2-mediated activation in chronic prurigo as well as pre- and post-treatment levels of histamine and eicosanoids in atopic dermatitis (28, 29). However, microdialysis does not assess the total tissue concentration of histamine or other mediators. In live human volunteers, microdialysis is limited to the use of only approved drugs and agents. Additional limitations are that commercially available microdialysis equipment is expensive and lacks flexibility in its application (30).

The human skin explant model has been utilized to investigate the short-term effects of cytokines and irritants on skin while avoiding the ethical problems of *in vivo* stimulation studies with non-approved agents (31). Under appropriate conditions, skin explants may remain viable for 14 days at 37 °C (32). Intact skin explants generated from biopsies have been utilized to examine the role of cytokines in the non-histaminergic pruritus pathway induced by TRPV1-, TRPA1-and PAR2-agonists (33). In a study by Tausk and Undem, human skin fragments of various sizes were utilized to examine histamine release induced by substance *p* and stem cell factor (SCF) (34). Though, the applicability of the findings of these 2 studies to other mast cell activation pathways remained unknown. Another study sought to investigate histamine release in response to FcɛRI, and MRGPRX2 activation. However, the method of skin processing induced high levels of spontaneous histamine release (35). These results suggest that intact skin explants could serve as an alternative method for assessing mast cell activation.

This “Methods” paper describes the development and application of an intact skin explant model to study human skin mast cell activation. In brief, discarded surgical skin specimens were exposed to various conditions to measure spontaneous histamine release. Subsequently, the specimens were stimulated with well-characterized ligands and inhibitors of FcεRI and MRGPRX2 signaling. Residual histamine (RH) content was quantified in specimens incubated overnight in 1.6% perchloric acid. Finally, total skin histamine content (TSHC) was determined by adding spontaneous, stimulated, and residual histamine. Among the benefits of this method are the limited processing of skin specimens and relatively short time to completion (less than 24 h).

## Materials and equipments

### Skin specimens

**Figure 1:**
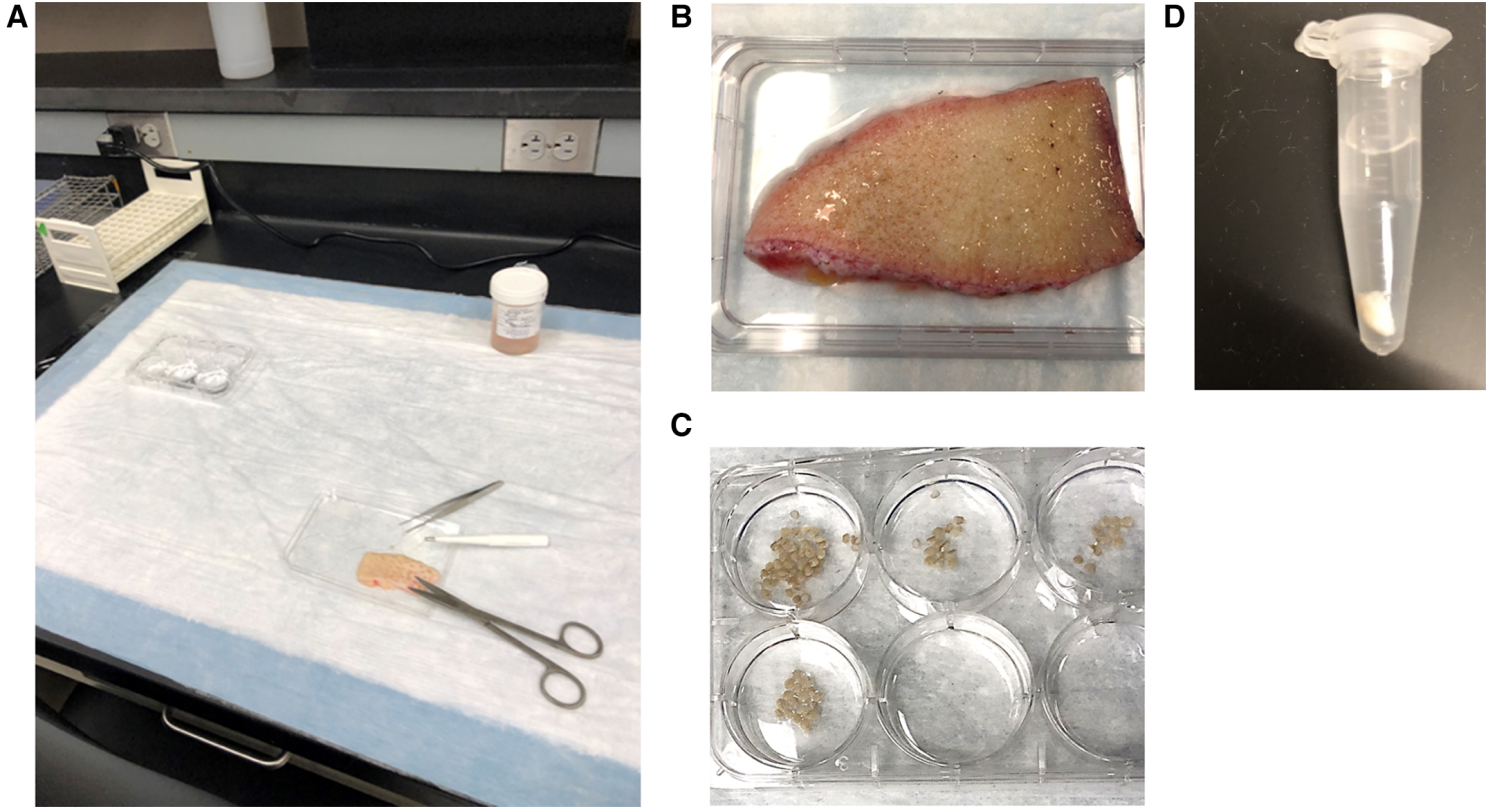
(**A**) bench set up for skin explant experiments. (**B**) Skin is placed on inverted lid of a 6-well culture plate which allows for the collection of any buffer that is released from the skin and provides a stable surface for the performance of punch biopsies. (**C**) Punch biopsies are immediately placed in wells containing PAG before being transferred to microcentrifuge tubes filled with PAGCM and/or inhibitors. (**D**) Following stimulation, biopsies are transferred to perchloric acid. Shown here is a 4 mm punch in 1 ml of 1.6% perchloric acid.

– De-identified, non-diseased human skin tissue without adipose layer from abdominal or breast surgeries was provided by the National Disease Research Interchange (NDRI) (Figure 1). Samples were provided from only adult individuals without a history of cancer or history of chemotherapy or radiation within the previous 5 years.
– De-identified, non-diseased human skin tissue was provided from adult plastic and reconstructive surgeries at Johns Hopkins Bayview Medical Center. Occasionally, these samples included the adipose layer which was removed prior to performing biopsies. These samples were not prescreened for cancer or radiation history, and age and gender were not provided.
– Consent was obtained by surgeons for the use of tissue for research.
– This study was determined to be IRB-exempt using their questionnaire because it fell within the category of “Not Human Subjects Research (NHSR)/Quality Improvement (QI).” Proof of this exemption was formally obtained from the IRB in the form of a letter as required by NDRI before shipping skin.

### Skin procedure

– Sterilized fine splinter forceps 4.5 in. forceps (Medline)
– Sterile smooth, straight, stainless steel 5-1/2 in. scissors (Skylar Instruments)
– Sterile disposable OR Grade 2.5 mm and 4 mm punches (Acuderm)
– 6-well culture plate (Costar® 6-well Clear TC-treated Multiple Well Plates, Individually Wrapped, Sterile)
– Microcentrifuge tubes 1.5 ml
– Eppendorf Pipettes 10 µl, 100 µl, 1,000 µl
– TipOne pipette tips 10 µl, 100 µl, 1,000 µl
– Nitrile gloves
– Incubator
– Micro-Centrifuge (Labnet Prism C2500-R)
– Histamine autoanalyzer
– Autoanalyzer sample cups
– Orbital shaker
– Sharps Container
– Ethanol 70%

### Reagents

– Ultrapure water, Cayman Chemical
– Polyclonal goat anti-IgE, courtesy of Dr. Robert Hamilton, DACI Laboratory, Baltimore, MD
– Acalabrutinib (ACP-196), Cayman Chemical
– Compound 48/80, Sigma-Aldrich
– QWF [Boc-Gln-D-Trp(Formyl)-Phe-OBzl], TOCRIS
– Pertussis Toxin (PTX), Sigma-Aldrich
– PIPES-albumin-glucose (PAG) buffer, prepared in-house. PAG consists of 25 mM PIPES [Piperazine-N,N′-bis(2-ethanesulfonic acid)], 110 mM NaCl, 5 mM KCl, 0.1% glucose, and 0.003% human serum albumin (HSA)
– PAGCM, prepared in-house. PAGCM consists of PAG supplemented with 1 mM CaCl_2_ and 1 mM MgCl_2_.
– PAG-EDTA, prepared in-house. PAG-EDTA consists of PAG supplemented with 0.1 mM EDTA (ethylenediamine N, N, N′, N′- tetraacetic acid)
– Perchloric acid (HCLO_4_) 1.6%, prepared in-house on the day of the experiment by diluting stock supply (HCLO_4_ 61%) with deionized water.

## Stepwise procedures

### Preparation of reagents

– PAG and PAGCM were removed from fridge and allowed to come to room temperature (RT). The incubator was warmed to 37 °C.
– Reconstitute stimuli and inhibitors if necessary. *Note:* Compound 48/80 powder was reconstituted to a concentration of 10 mg/ml using ultrapure water. All other commercial reagents were reconstituted per manufacturer instructions. Stock concentrations of all reagents were further diluted in PAGCM.
– Microcentrifuge tubes were filled with PAGCM (with or without inhibitors) as indicated below, and placed in the incubator to warm. *Note:* Explants used for inhibition conditions are exposed to the inhibitors during the spontaneous HR incubation and stimulation.
– The total volume of buffer in which explants were incubated was 100 µl for anti-IgE and its inhibitors, 75 µl for compound 48/80 and its inhibitors, and 75 µl for experiments that examined anti-IgE and compound 48/80 responses in the same donor.
– For each stimulus and inhibitor, prepare [2×] concentration (working concentration). This will be combined with an equal amount of buffer (or another reagent) in each microcentrifuge tube to create the final concentration at the appropriate volume. For example, 50 µl of 6 ug/ml [2×] anti-IgE was added to 50 µl of PAGCM to make 100 µl of 3 µg/ml [1×] anti-IgE.
  • For inhibition experiments, the same approach was taken. For example, 50 ul of 5 µg/ml [2×] acalabrutinib was added to 50 µl of 6 µg/ml [2×] anti-IgE to make 100 µl of 2.5 µg/ml [1×] acalabrutinib + 3 µg/ml [1×] anti-IgE.
– All HR incubations were performed in duplicate or triplicate. Separate tubes were designated for PGD_2_ measurement in duplicate.
– Prepare microcentrifuge tubes for spontaneous histamine release before processing the skin/performing biopsies.
– Prepare stimulation tubes while explants are incubating for spontaneous histamine release.
– All prepared microcentrifuge tubes were pre-warmed to help maintain consistent temperature of specimens.

### Skin stimulation procedures

**Figure 2:**
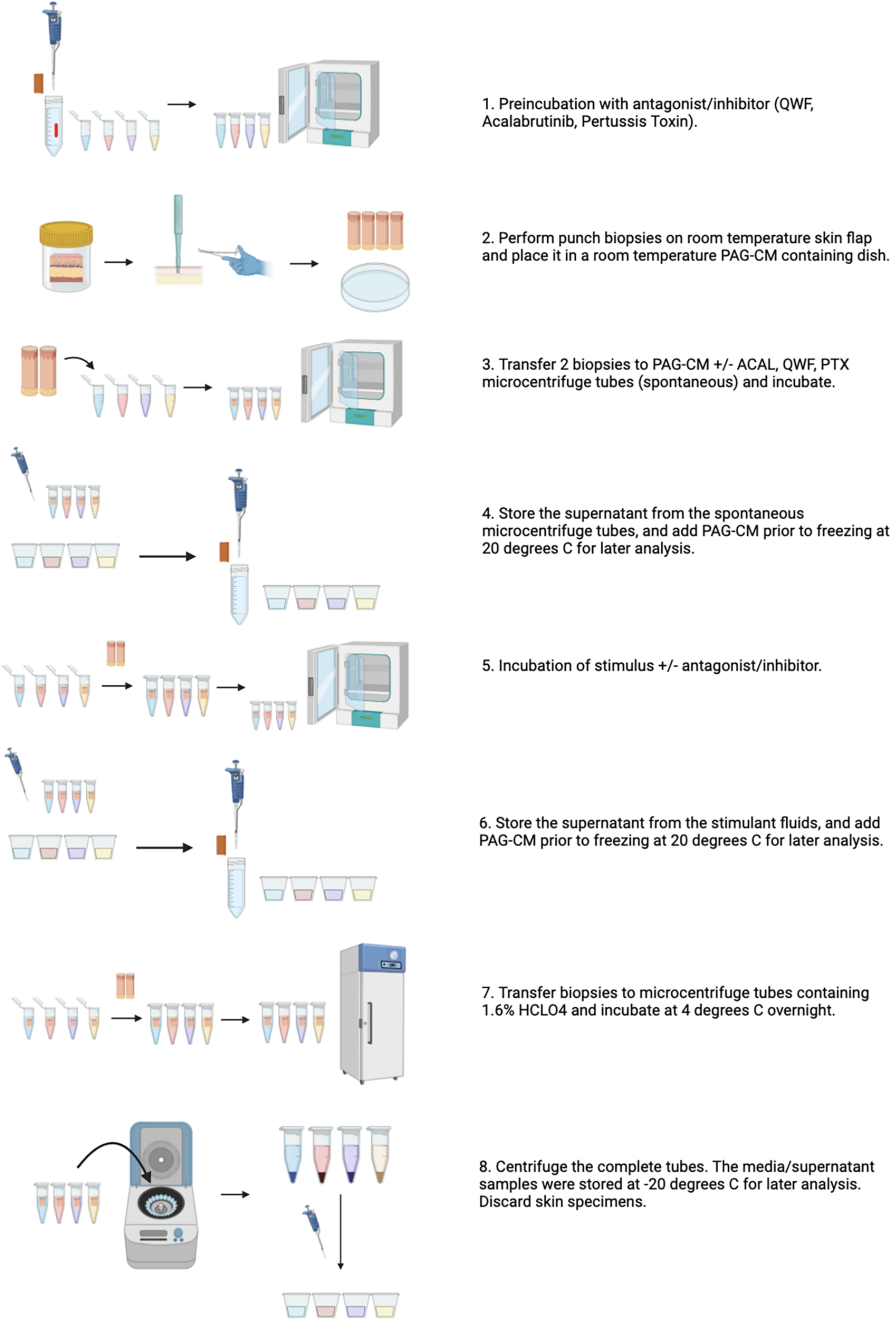
Overview of the skin explant model to study mast cell activation and inhibition. Created in Biorender.com.

– An overview of the explant stimulation and inhibition procedure is provided in Figure 2.
– NDRI skin segments, which were a minimum of 5 × 5 cm, arrived within 24 h of harvest on an ice pack in a sterile specimen container filled with phosphate buffered saline (PBS) and antibiotics.
– Specimen container was removed from shipping box or refrigerator and set on benchtop for 1 h to allow skin to reach RT.
– Specimens were divided into equal size samples using skin biopsy punches. *Note:* Experiments were initially conducted using both 2.5 mm punches and 4 mm punches. It was felt that using two 2.5 mm punches in each microcentrifuge tube produced more consistent results between duplicate and triplicate conditions.
– Wells of a 6-well culture plate were filled with RT PAG. Skin was set on the lid from 6-well culture plate for stabilization. Specimens were biopsied using a 2.5 mm or 4 mm punch. Care was taken to avoid taking biopsies from stretch marks. Biopsies were transferred to wells containing RT PAG as they were obtained to prevent specimens from drying out.
– Punch biopsies were placed in pre-warmed microcentrifuge tubes containing PAGCM with/without inhibitors.
– Biopsies were incubated at 37 °C for 1 h to assess spontaneous HR. In the case of QWF and PTX, the spontaneous HR incubation time was the same as the duration of pretreatment with the inhibitor (60 min). This incubation was shortened to 30 min for experiments involving acalabrutinib. Thus, for anti-IgE experiments that involved acalabrutinib, spontaneous HR was measured after 30 min for all samples.
– Following the spontaneous HR incubation, biopsies were transferred to stimulation tubes using designated forceps for each experimental condition to avoid cross-contamination (i.e., using inhibitor forceps on stimulation-only samples).
– Spontaneous HR buffer was transferred to histamine cups and brought to a volume of 1 ml with PAGCM. This was frozen (−20 °C) for later spontaneous histamine release assessment.
– Stimulation tubes were returned to incubator for 1 h to assess stimulated histamine release (see Results section for data supporting the decision to use a stimulation time of 1 h). Following stimulation, biopsies were transferred to designated tubes filled with 1 ml of RT 1.6% perchloric acid. Stimulation HR buffer was transferred to histamine cups and brought to a volume of 1 ml with PAGCM before being frozen (−20 °C).
– Following overnight incubation in perchloric acid at 4 °C, samples were centrifuged at 12,000 rpm for 4 min and the supernatant was harvested for histamine release. Residual histamine (RH) in samples was either analyzed immediately (along with stored stimulated and spontaneous histamine release samples from the day before) or stored at −20 °C until later analyzed.

### Processing and stimulation of fresh surgical skin specimens

– Fresh surgical skin specimens, approximately 2 × 2 cm, were provided by JHBMC, within 2–4 h of surgery, in a sterile specimen container at room temperature without buffer or media.
– Specimens were placed in RT PAG for one hour or washed in EDTA (followed by increasing concentrations of calcium) at the start of the experiment. For the EDTA wash, the intact surgical specimen was placed in PAG/0.1 mM EDTA for 20 min, then transferred to PAG for 20 min, then a 1:1 mix of PAG + PAGCM (0.5 mM CaCl_2_) for 20 min, then PAGCM (1 mM CaCl_2_) for 15 min. Each wash was performed in a 6-well plate on an orbital shaker (65 rpm) in a volume of 3 ml. Following the final wash, 2.5 mm punch biopsies were obtained and assessed for spontaneous, stimulated, and residual histamine as described above.

### Mediator quantification

We measured spontaneous, stimulated, and residual histamine (RH) of 2.5 mm biopsy pairs or single 4 mm biopsies using a fluorometric autoanalyzer (36). Spontaneous histamine release (HR) was assessed following incubation in buffer alone for 1 h at 37 °C. Stimulated HR was determined for various concentrations of MC stimuli ± their respective inhibitors or antagonists at various time points at 37 °C. Inhibitors and antagonists were added during the spontaneous incubation and again during the incubation with the stimuli. RH content was extracted by incubating biopsies overnight at 4 °C in 1.6% perchloric acid. Total skin histamine content (TSHC) was calculated by adding HR and RH content for each biopsy pair. All results were expressed as a percentage of TSHC. Prostaglandin D_2_ (PGD_2_) was assessed using a Cayman Chemical prostaglandin D_2_ ELISA (Item No. 512031).

### Statistical analysis

All summary data are presented as mean ± SD. Statistical analyses were performed with GraphPad Prism (version 10.1.2) using Wilcoxon signed rank test or Mann Whitney test for paired and unpaired samples, respectively. A Spearman rank correlation test was used to assess the relationship between 2 variables. A Kruskal-Wallis test or an analysis of variance (ANOVA) with Tukey's multiple comparison test was performed to determine whether there was significant variation between 3 or more variables. A *p* value <0.05 was considered significant.

## Results

### Total skin histamine content

**Figure 3:**
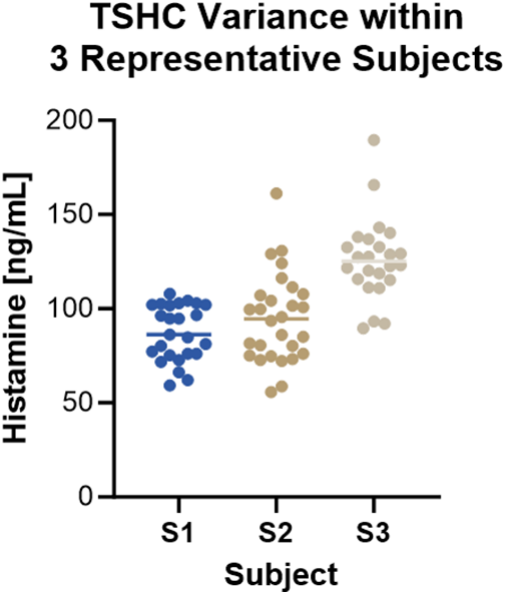
Total skin histamine content varied between and within individual donors. Data is shown for 3 subjects. Each circle represents a pair of 2.5 mm punch biopsies. Mean THSC was 87.12 ± 14.8 ng/ml, 94.80 ± 23.73 ng/ml, and 126.1 ± 1.69 ng/ml for subjects S1, S2, and S3, respectively. Kruskal-Wallis test, *p* < 0.0001.

We determined the total skin histamine content of 2.5 mm biopsy pairs (Figure 3) and single 4 mm biopsies (Supplementary Figure S1) by adding the spontaneous, stimulated, and residual histamine of each sample. For both conditions, we observed that the total skin histamine content of biopsies varied between and within individual donors. A strong correlation was found between tissue weight, which also varied, and total skin histamine content; however, variance in total skin histamine content was still observed after histamine content was corrected for tissue weight (Supplementary Figure S1). In a prior study by Eady et al, light microscopy revealed wide variations in mast cell counts between different sections from the same biopsy specimen, confirming that mast cells are unevenly distributed even within the same biopsy specimen (37). Therefore, we hypothesize that differences in weight and mast cell distribution between biopsies are responsible for the variance that we observed in total skin histamine content. There were few studies to draw upon as a guide for the expected total histamine content of skin. One study reported that the histamine content of healthy skin was 15.7 ± 3 (mean ± 2 SEM) ng/mg. Notably, that study used a process of boiling minced skin for 5 min to extract tissue histamine (38). Using overnight perchloric acid digestion at 4 °C to extract residual histamine, we found that the total histamine content of most of our skin biopsies (when adjusted for weight) fell within this range while others exceeded this range.

### Spontaneous histamine release

**Figure 4:**
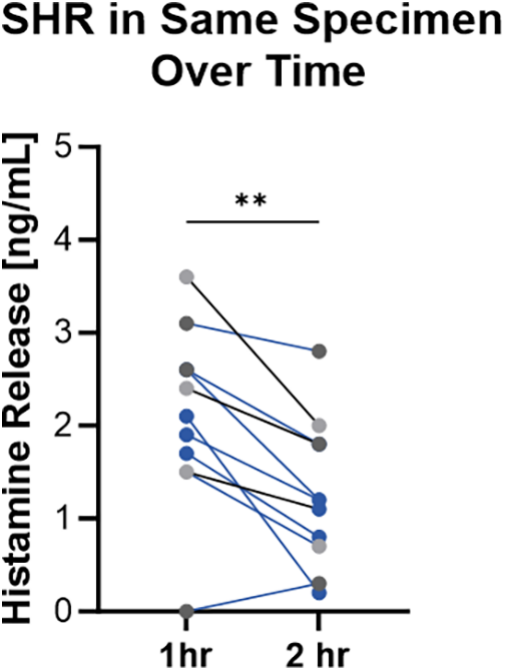
Spontaneous histamine release varied over time and between donors. Pairs of 2.5 mm punches were incubated at 37  °C. Buffer was harvested and replaced at 1 h and 2 h. Data shown are histamine release during those time periods from 3 donors with number of samples per donor ranging from 2 to 4. Wilcoxon paired sign rank test, *p* = 0.0029.

**Figure 5:**
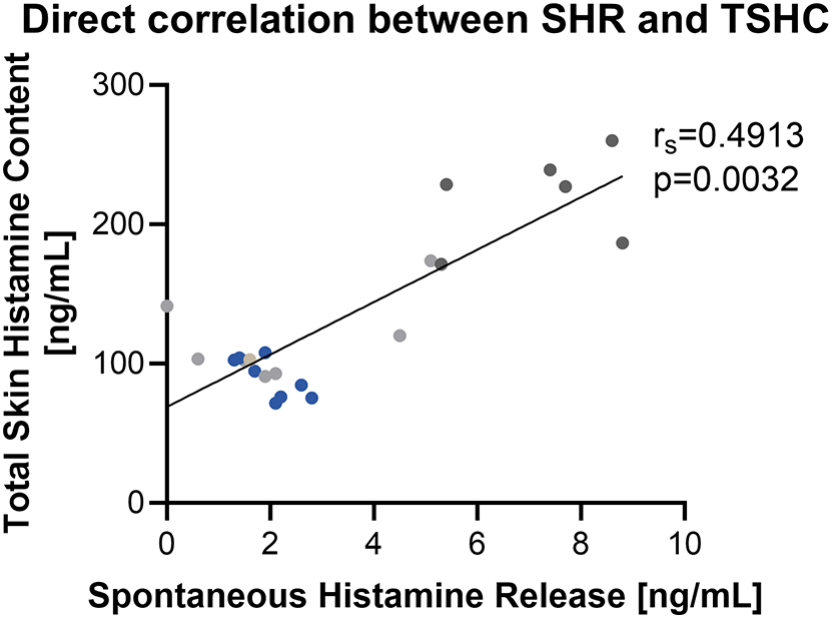
Spontaneous histamine release is directly correlated with total skin histamine content at physiologic temperature. Data shown are histamine release during those time periods from 3 donors with number of samples per donor ranging from 6 to 10 paired 2.5 mm punches. Each color represents a different donor.

We observed variability in net spontaneous histamine release between subjects and within subjects. The highest levels of spontaneous histamine release were noted initially following the skin biopsy procedure and values decreased over time (Figure 4). To further understand the variability in raw spontaneous histamine release between samples, we examined the relationship between spontaneous histamine release and total skin histamine content. We observed that spontaneous histamine release was directly correlated with total skin histamine content (Figure 5, Supplementary Figure S2). Despite the variability in raw histamine release, the initial spontaneous histamine release for NDRI skin specimens as a percentage remained low, only exceeding 2% of total skin histamine content on rare occasions. To standardize our results, we report histamine release as a percentage of total histamine content in the sections that follow.

### Stimulated mediator release

**Figure 6:**
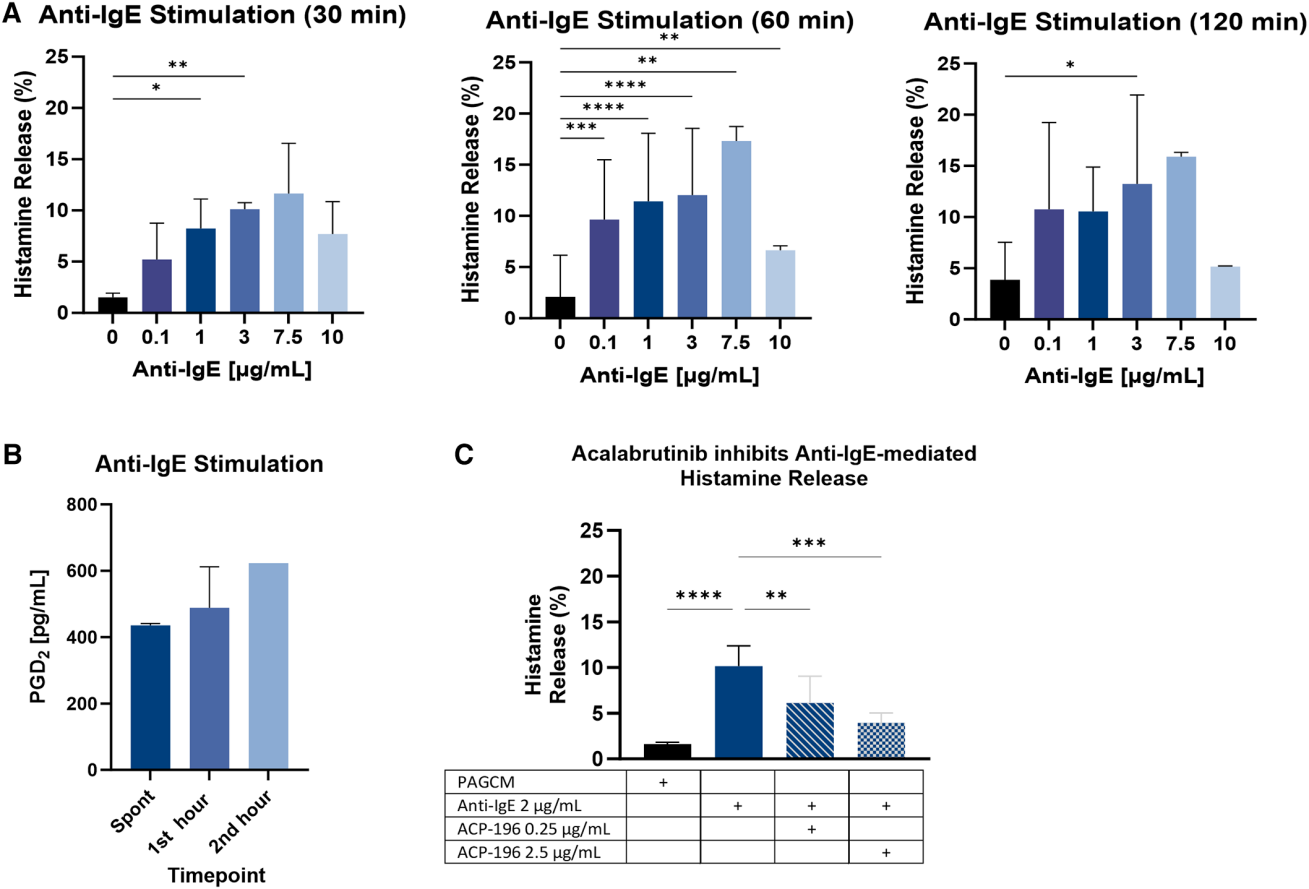
IgE-mediated mediator release. (**A**) The kinetics of explant histamine release following stimulation with polyclonal goat anti-IgE are shown for 30 min, 60 min, and 120 min. Paired 2.5 mm punches were examined in triplicate. Data is shown for the same 3 donors for each time point. (**B**) PGD_2_ production was measured from 4 mm punches from 2 subjects at baseline (1 h before stimulation) and at 60 min and 120 min following stimulation with 1 µg/ml anti-IgE (note: results reflect PGD_2_ produced during the 1st and 2nd hour following stimulation, not cumulative production). (**C**) Inhibition of IgE-mediated histamine release by acalabrutinib is shown for 3 donors. Paired 2.5 mm punches were examined in duplicate. Samples were incubated with acalabrutinib (ACP-196) for 30 min before stimulation and again during stimulation with anti-IgE. Results for histamine kinetics and acalabrutinib inhibition were analyzed using ANOVA with Tukey’s multiple comparison test. Only significant *p* values are shown. *p < 0.05, **p < 0.005, ***p < 0.0005, ****p < 0.0001.

We established the kinetics of IgE-mediated histamine release for this model by quantifying histamine release at 2, 5, 10, 20, 30, 60, and 120 min in triplicate (not shown). While histamine release could be detected as soon as 5 min in response to the highest concentration of anti-IgE, it was not observed in all replicates. This may have been due to variation in the diffusion of anti-IgE into the tissue during these shorter time periods as well as mast cell distribution. Results of anti-IgE mediated histamine release were more consistent for the 30-, 60-, and 120-min stimulation periods (Figure 6A). It was previously reported that, depending on the donor, optimal histamine release for purified skin mast cells was obtained using an anti-IgE concentration of either 1 µg/ml or 3 µg/ml (39). We found no significant difference between the results for these concentrations. Using 4 mm punches, we confirmed the ability to detect mast cell-derived PGD_2_ following anti-IgE stimulation (Figure 6B).

To determine whether anti-IgE mediated histamine release could be inhibited by a specific inhibitor of IgE signaling, we pretreated samples with the BTK inhibitor acalabrutinib. We found that the optimal inhibitory concentration of acalabrutinib in this model was 2.5 µg/ml (Figure 6C). Pertussis toxin also inhibited anti-IgE-mediated histamine release (Supplementary Figure S3). Our results are in line with a prior study which showed that incubation with pertussis toxin for 4 h at 37 °C inhibited anti-IgE mediated histamine release from dispersed human skin mast cell cultures by 63.3 ± 8.2% (40). In contrast, an earlier study demonstrated that pertussis toxin failed to inhibit anti-IgE (3 µg/ml) mediated histamine release from dispersed human skin mast cells (41).

**Figure 7:**
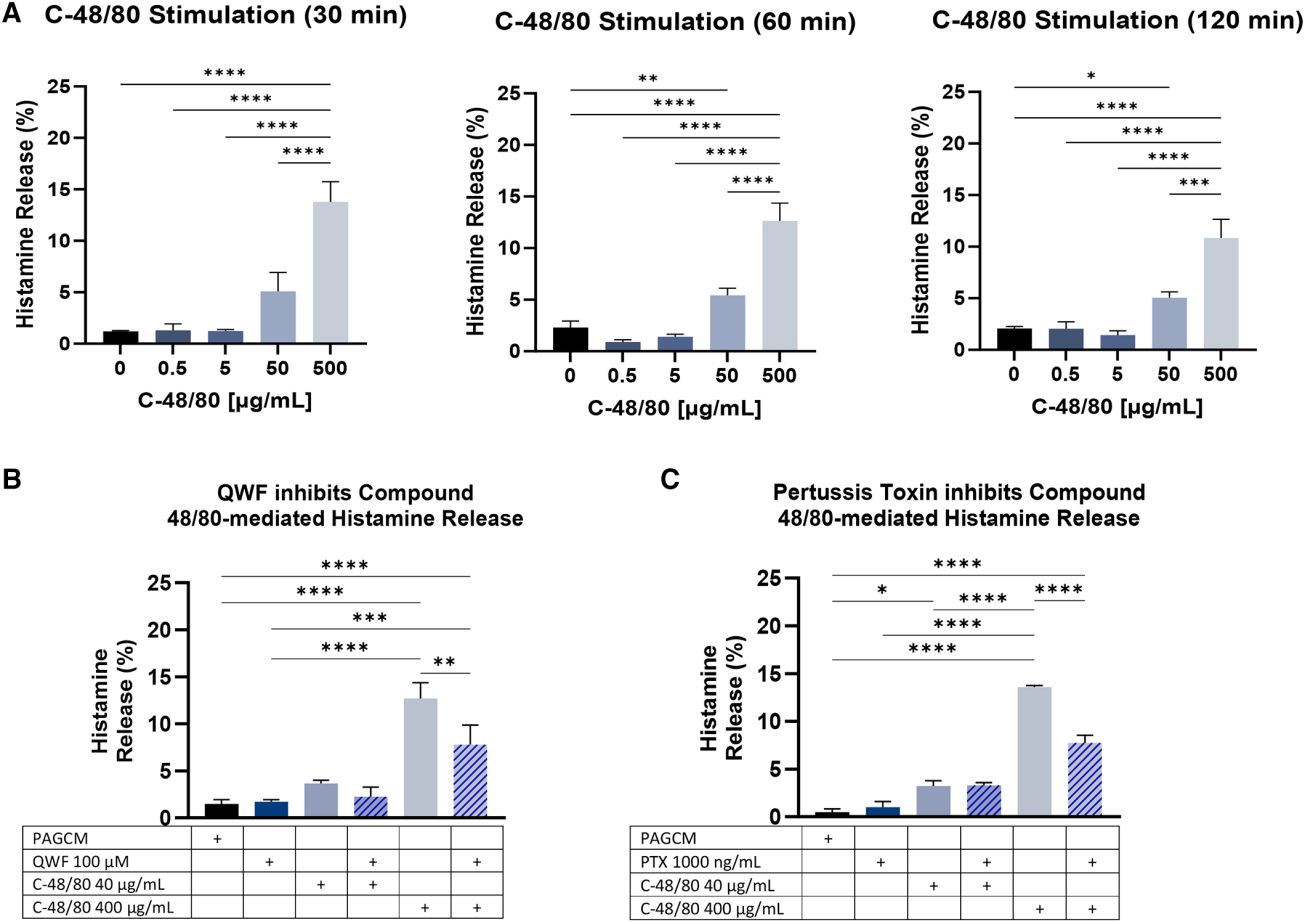
Compound 48/80-mediated histamine release. (**A**) The kinetics of explant histamine release following stimulation with compound 48/80 (C-48/80) are shown for 30 min, 60 min, and 120 min. Paired 2.5 mm punches were examined in duplicate. Data is shown for the same 3 donors for each time point. (**B**) QWF at 100 µM concentration shows inhibition of compound 48/80 mediated histamine release. Data from 2 donors in duplicate (paired 2.5 mm punches). (**C**) Inhibition of compound 48/80 mediated.

Based on our anti-IgE results, we next examined the kinetics of compound 48/80-mediated histamine release at 30, 60, and 120 min. Results were similar across these 3 time points (Figure 7A). To determine whether compound 48/80-mediated histamine release could be inhibited in our model, we pretreated samples with QWF (Figure 6B) or pertussis toxin (Figure 6C). Although MRGPRX2 antagonists have been developed for clinical use and are in phase I and phase II trials, we did not have access to these agents. Therefore, we utilized QWF which has been reported to be an antagonist of MRGPRX2 (42). QWF and pertussis toxin have been shown to inhibit compound 48/80-mediated degranulation in human mast cell lines (26, 42) and human cord blood-derived mast cells (43), and we observed that these agents inhibited histamine release in our explant model.

**Figure 8:**
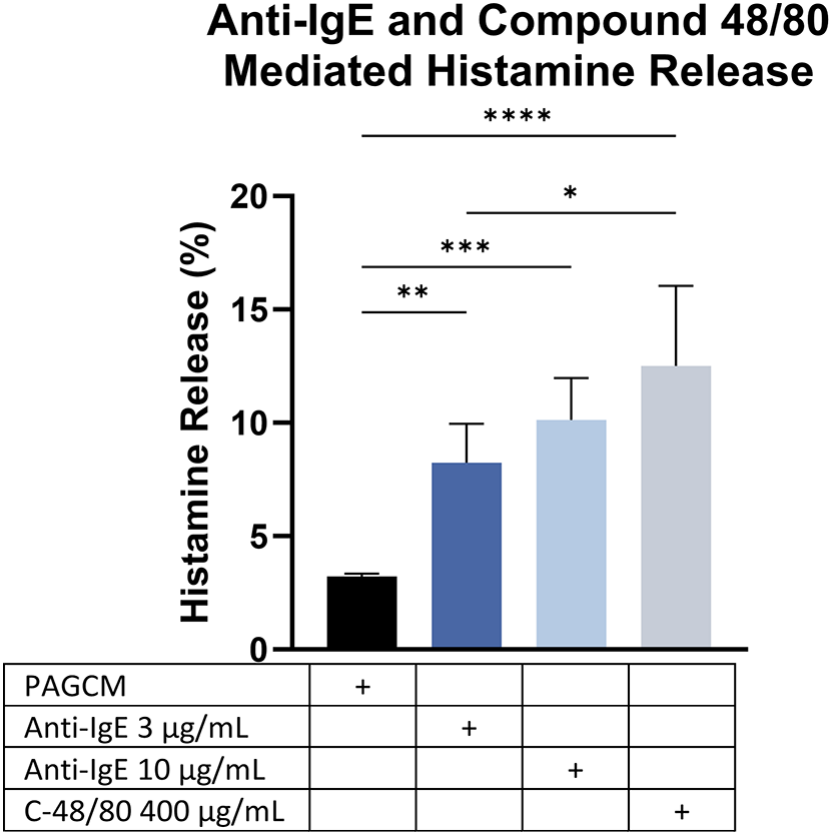
Comparison of IgE-mediated and compound 48/80-mediated histamine release within the same individuals. Data are shown for 3 donors. Paired 2.5 mm punches were examined in duplicate. ANOVA with Tukey's multiple comparison test. *p < 0.05, **p < 0.005, ***p < 0.0005, ****p < 0.0001.

After establishing the kinetics of histamine release, we examined anti-IgE- and compound 48/80-mediated histamine release in the same donors. Utilizing a stimulation period of 1 h, we found greater histamine release in response to compound 48/80 than with anti-IgE (Figure 8). Our results are in contrast with previous studies which showed that cultured skin-derived MCs were less responsive to MRGPRX2 activation than Fc*ε*RI-aggregation due to both acute and chronic inhibition by SCF in the culture media (44, 45). Furthermore, skin mast cells in culture undergo several non-synchronized modulations. For instance, tryptase and chymase expression strongly decline over time (46). There is also a marked increase in Fc*ε*RI surface expression and Fc*ε*RI-mediated histamine release (increasing from ≈15.5% to ≈60%) during the culture period (46).

### Results from fresh surgical specimens

**Figure 9:**
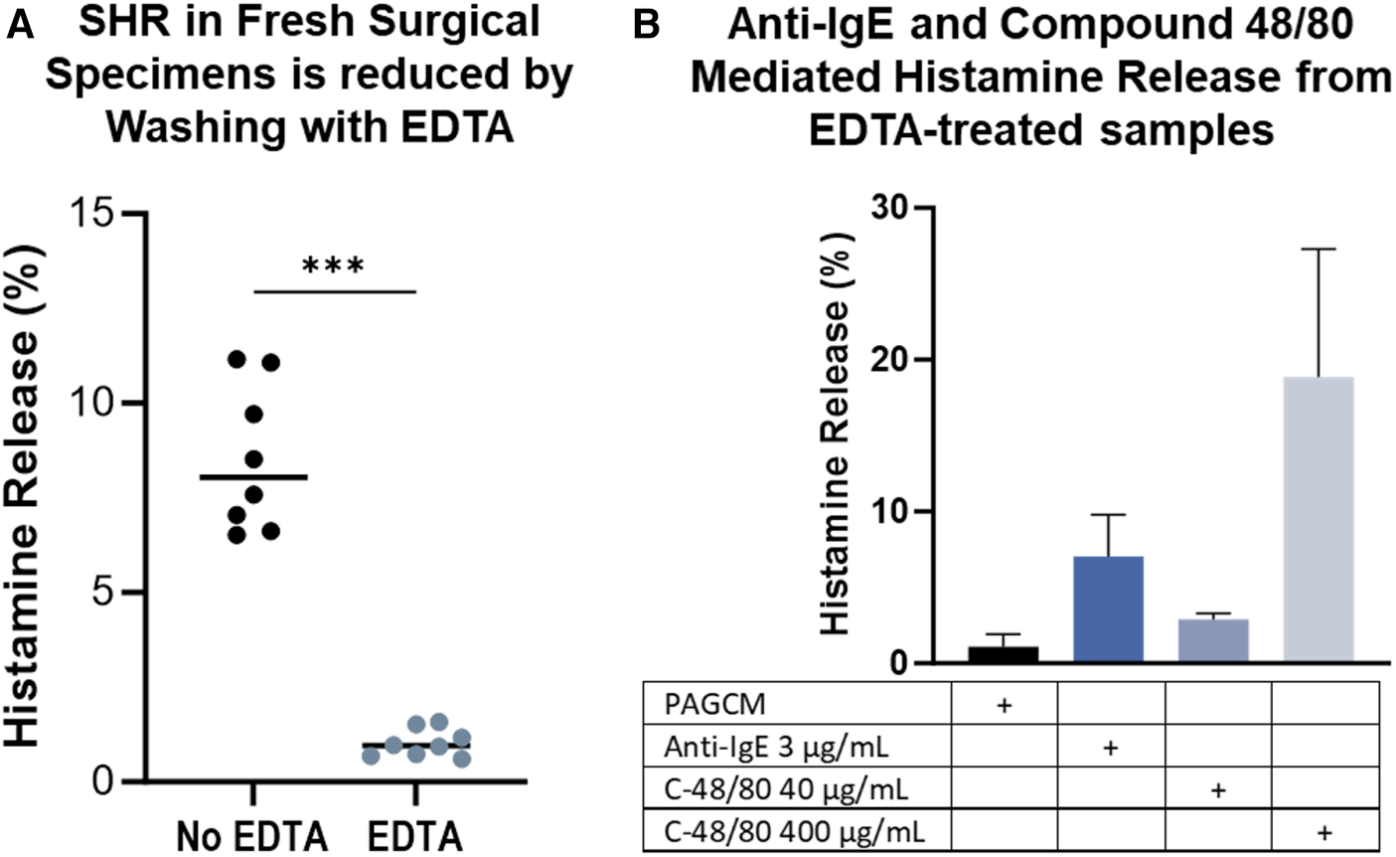
EDTA treatment minimizes spontaneous histamine release from specimens stimulated the same day as surgery while preserving stimulated release. Data is shown for 2.5 mm biopsy pairs from the same donor, same surgery day. (**A**) Spontaneous histamine release is reduced by EDTA. Mann Whitney test, *p* = 0.0002. (**B**) Samples were washed in EDTA followed by increasing concentrations of calcium at room temperature before assessing spontaneous and stimulated histamine release. Data shown for the same subject in duplicate.

We examined histamine release from fresh surgical specimens obtained on the same day and noted that spontaneous histamine release was markedly elevated. In some cases, spontaneous histamine release exceeded 60% within the first hour following biopsy, but this decreased over time in the same specimens (Supplementary Figure S4). We questioned whether the trauma of the procedure or skin processing caused this steep rise in spontaneous histamine release. We found that warming of the skin did not have an appreciable impact on spontaneous histamine release. Elevated spontaneous histamine release also occurred despite incubating specimens at room temperature in PBS/1% BSA instead of a high calcium-containing buffer (Supplementary Figure S4). Thus, we hypothesized that the combination of trauma and existing calcium in the skin contributed to high spontaneous histamine release. Incubating the skin specimen in EDTA and washing in increasing concentrations of calcium reduced spontaneous histamine release to 1.03 ± 0.37% while allowing detection of histamine release via anti-IgE and compound 48/80 (Figure 9).

## Discussion

This “Methods” paper describes the development and application of an intact skin explant model to study human mast cell activation. This method allows for the comparison of dose responses to distinct mast cell stimuli and their antagonists or inhibitors even between different donors.

Prior studies of mast cell activation in humans have utilized well-characterized commercially available cell lines, mast cell cultures derived from tissue or CD34+ progenitor cells, *in vivo* skin testing, skin chambers, and microdialysis. However, each of these methods has drawbacks. The applicability of cell cultures to human skin disorders may be limited as mast cells are no longer in a natural state. The process of generating mast cell cultures from tissue or progenitor cells can be costly and time consuming. *In vivo* skin testing assesses visible wheal and erythema formation in response to stimuli, but alone does not provide a means for direct sampling of released mediators. In contrast, skin chamber studies and microdialysis can assess levels of endogenous as well as exogenous substances or drugs administered to patients. Traditional skin chamber studies have a long time course for blister formation (up to 2 h), a tendency for blisters to coalesce, and occasional ecchymoses (47). Newer negative pressure devices have a shorter time course of blister formation due to the combination of heat and higher pressure. Mediator assessment is still limited by the number of blisters that can be formed and the size of the collection chambers used (48). *In vivo* microdialysis studies require the use of topical anesthetics to limit discomfort while *ex vivo* studies require access to large sections of tissue. In addition, financial cost may limit widespread use of microdialysis for both *in vivo* and *ex vivo* studies. Both *in vivo* microdialysis and skin chamber studies are also limited to the use of only approved pharmacologic therapies in inhibition studies.

The advantages of our skin explant model include: the avoidance of regulatory and safety issues with studies of live patients, the use of small skin samples to assess mast cell responses, and a quick turnaround time for experiments. Another benefit of this skin explant model is no processing or manipulation of the skin allowing receptors and granules to remain intact. Compared to culturing and cloning HMC1 and LAD2 cells, this skin procedure takes less than 2 days to complete and involves less manipulation. Although the HMC1 cell line may have a faster growth rate than other cell lines, it is inadequate in studying degranulation of mast cells (26). Our method provides the advantage of closely mimicking the *in vivo* immunological mast cell response.

Given the variability in histamine content of biopsies between and within the same donors, our model requires analysis of histamine release as a percent of the total histamine content for each sample. Using designated specimens to serve as “representative” specimens for spontaneous release or total histamine content for an entire experiment could result in misleading results (mistaking spontaneous release for stimulated release). We encountered this problem when we began to develop this model as the first specimens that we received were from fresh surgical specimens which initially had a high level of spontaneous histamine release at baseline.

Moreover, beta-hexosaminidase assays are commonly performed in place of histamine assays in determining degranulation in mast cells. Although we did not measure beta-hexosaminidase in our study, we anticipate that our model can produce similar beta-hexosaminidase results based on prior studies that demonstrate a correlation between beta-hexosaminidase and histamine assays (15, 29, 49, 50).

In the development of this explant model, efforts were made to minimize spontaneous histamine release. It is important to distinguish the receptor-mediated mast cell histamine release from spontaneous degranulation. Due to the high level of spontaneous histamine release from fresh surgical samples initially, we were initially unable to distinguish stimulated release from spontaneous release during the stimulated period (Supplementary Figure S4). However, the protocol we developed for fresh same-day specimens reduced spontaneous release while maintaining the ability to detect stimulated release (Figure 9).

Using this model, the magnitude of stimulated histamine release may vary depending on the agent used or pathway interrogated. We found that compound 48/80 produced higher histamine release than anti-IgE activation (Figure 7). Our results are in contrast with previous studies which showed that cultured skin-derived mast cells were less responsive to MRGPRX2 activation than FcεRI-aggregation due to both acute and chronic inhibition by SCF (44, 45). SCF has also been shown to acutely enhance Fc*ε*RI-mediated mast cell degranulation while chronic exposure may reduce Fc*ε*RI-mediated degranulation (51). In one study, skin mast cell Fc*ε*RI-mediated histamine release was shown to increase nearly fourfold when mast cells were cultured in SCF + IL-4 for up to 16 weeks (46). There was also a corresponding increase in Fc*ε*RIα surface expression, which the authors speculated was due to constant exposure to IL-4. Thus, an advantage of our model is that the response to Fc*ε*RI and MRGPRX2 activation may be more consistent with the natural state as our method does not require addition of SCF or cytokines for culture.

As expected, the kinetics for stimulated histamine release in our explant model were notably longer than for isolated cell suspensions and tissue homogenates. For instance, in one study, anti-IgE-mediated histamine release from mast cells in skin tissue homogenates reached 14.2% for normal controls following 20 min incubation with the optimal dose of anti-IgE (52). In our model, median stimulated histamine release for 1 µg/ml anti-IgE was only 4.66% at 20 min, but rose to 15.75% at 1 h with this same dose (one donor, in triplicate). Thus, our results are in line with those of skin homogenates when a longer stimulation period is conducted. Our work demonstrates that, under optimal conditions, intact explants may serve as a useful model for assessing human skin mast cell function without enzymatic digestion or mincing the skin.

High levels of spontaneous histamine release have been observed in studies utilizing tissue homogenates. Veien et al. utilized dispersed mast cell suspensions isolated from neonatal foreskins to understand the mechanisms of histamine and tryptase release induced by vancomycin, morphine, and atracurium (53). They found that these drugs caused the release of both histamine and tryptase, and that this activation could be inhibited by blocking phospholipase C and phospholipase A2. However, data was excluded from samples with spontaneous histamine release exceeding 15%, which the authors attributed to cell lysis during processing (53). In another study, Ruzicka and Gluck compared mast cell responses in atopic dermatitis skin lesions and healthy control skin using tissue homogenates which were generated by mincing fresh biopsies approximately 0.5 cm^2^ in size (54). The authors found that atopic skin released twice as much histamine in response to anti-IgE than healthy skin whereas compound 48/80-mediated release was almost identical between the two groups. However, the mean spontaneous histamine release was markedly elevated at 31.6% ± 4.2% (mean ± SEM) for healthy controls and 25.9% ± 4.0% for the patients with atopic dermatitis (54). The authors postulated that the preparation of sections of the firm skin tissue led to the damage of mast cells. If this were indeed the cause, it is possible that these high levels of spontaneous histamine release could have been avoided with our intact skin explant model.

A study by Clegg et al. sought to characterize histamine release from fresh human foreskin slices in response to anti-IgE, compound 48/80, and other synthetic secretagogues, as well as the inhibitory effects of salbutamol and sodium cromoglycate (35). The authors utilized a tissue chopper to generate 200 µm thick fresh foreskin slices which were then washed in 2 ml of Hanks' Balanced Salt Solution (HBSS) before the experiment. With this preparation method, the authors noted that spontaneous histamine release was high (ranging from 10 to 30%). The authors attempted to reduce spontaneous histamine release by washing the slices twice in a larger volume of HBSS (5 ml), allowing the slices to rest for 1 h, then washing for a third time before challenge. This washing procedure reduced spontaneous histamine release to 7.5 ± 0.5% but also reduced the skin slices' stimulated response to all secretagogues and increased the number of specimens failing to release any histamine during stimulation (35). In our model, we found that incubating fresh skin specimens in EDTA and washing in increasing concentrations of calcium reduced spontaneous histamine release while allowing detection of stimulated histamine release via anti-IgE and compound 48/80.

To date, few studies have utilized intact human tissue to study *ex vivo* mast cell degranulation. Of these studies, most have involved the use of microdialysis on large skin explants. *Ex vivo* microdialysis allows the use of nonapproved agents, however cost and access to large enough tissue limit its widespread use. Alternatively, Tausk and Undem examined net histamine release, induced by the MRGPRX2 ligand substance *p* and SCF, from small human neonatal foreskin and adult face and back skin fragments weighing between 9 and 48 mg. They found that large concentrations of substance *p* induce histamine release from skin mast cells, and that it was unlikely that these concentrations are reached physiologically during sensory nerve stimulation with capsaicin (34). Our model builds on this work and provides a more convenient and accessible alternative to microdialysis.

As our study entailed the use of de-identified surgical specimens, it is unknown whether donor characteristics influenced our results. These factors include atopic history and other medical history, medications administered before and during surgery, and age. With the exception of one NDRI donor, all skin specimens were from female donors. Thus, we were unable to assess whether there were sex differences in mast cell responses in this model. As our NDRI samples were exclusively either breast or abdomen, it is also unclear how our results would have differed had other body sites been evaluated.

Occasionally, there were NDRI surgical resections from which no appreciable histamine release could be elicited with anti-IgE or compound 48/80. We considered several possibilities for this finding including whether these individuals had received treatment with a drug that inhibited their mast cell activation. We also questioned whether these individuals had impaired mast cell responses at baseline. In a subset of the general population, basophil nonresponders or nonreleasers are well-described. In such individuals, blood basophils have poor histamine release in response to IgE-crosslinking with a polyclonal IgG antibody while other activation pathways remain intact (55). While this nonreleasing phenotype has also been observed in Syk-deficient human lung mast cells (56), this functional phenotype has not been described in human skin mast cells. Another possibility considered for the poor histamine response of these explants is that their mast cells perhaps degranulated in transit. However, analysis of the residual histamine content in these skin samples was comparable to that of those individuals who had demonstrable histamine release via FcɛRI and MRGPRX2 in our study. Nevertheless, given the wide variability in total skin histamine content between donors, we cannot conclude that nonresponsive specimens had not been partially depleted of histamine before arrival.

Our model specifically evaluated MRGPRX2 and Fc*ε*RI function of skin mast cells in discarded surgical skin specimens using well-studied reagents. The use of this explant model to investigate other agents could be limited by the diffusion capability of reagents in the skin. It is also possible that this model may not be applicable to the evaluation of mast cell histamine release from skin punch biopsies obtained directly from patients due to the inhibitory effects of lidocaine and epinephrine on IgE- and 48/80-induced mast cell histamine release (57–59). Additional work is required to optimize this model for fresh punch biopsies from patients.

The viability of skin mast cells is another potential limitation of this model. While other studies have reported skin viability up to 14 days (32), it is unclear whether mast cell responses will remain intact for that period of time. Data presented in this methods paper are from experiments performed on skin either the same day as or one day after surgery (NDRI). We have observed that histamine release can be detected 2 days after surgery in specimens stored in PBS+ antibiotics and 3 days after surgery in specimens stored in RPMI+ antibiotics (data not shown). Further investigations are required to determine the effect of time on mast cell viability and releasability in this model.

Our study characterized the kinetics of histamine release induced by anti-IgE and compound 48/80 in skin explants and the ability of acalabrutinib, QWF, and pertussis toxin to inhibit histamine release. Potential applications of our model include investigations of allergic diseases and evaluation of therapeutics under development to block these processes. This model can be also extended to examine levels of other mast cell mediators including tryptase, eicosanoids, and cytokines in response to activation of these pathways and whether other agents can effectively inhibit their release. However, this model has limitations when it comes to measuring cytokines and eicosanoids due to the production of these mediators of other cell types found in the skin. In addition, this model is not suited for interrogation of signaling pathways or molecular analysis requiring single cell suspension.

## Conclusion

The protocol reported here describes our methodology to successfully model human mast cell activation using intact skin biopsies. This model provides reproducible results and serves as an option to study possible stimuli and inhibitors for mast cell activation. In the future, this model may be extended to study altered mast cell function in various disease states, including primary mast cell disorders and chronic urticaria.

## Data Availability

The raw data supporting the conclusions of this article will be made available by the authors, without undue reservation.
