# Supplementary material for: Functional human skin explants as tools for assessing mast cell activation and inhibition
Source: Front Allergy. 2024 Mar 27;5:1373511. doi: 10.3389/falgy.2024.1373511 (PMC11004268; doi:10.3389/falgy.2024.1373511)
Supplement: Supplementary file 1 [file Datasheet1.docx]

**SUPPLEMENTARY FIGURES**


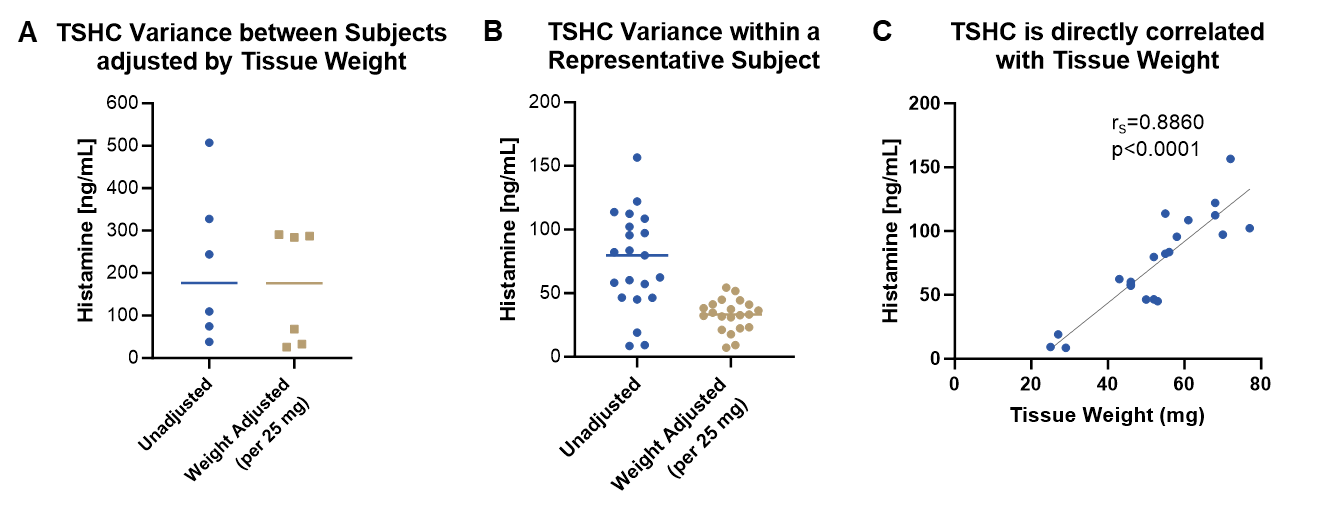


**Figure S1.** Total skin histamine content in 4 mm biopsies varied between and within individual donors. Data is shown for each punch biopsy from **(A)** 6 subjects and **(B)** 1 individual subject, with and without adjustments for tissue weight. **(C)** Total skin histamine content was directly correlated with tissue weight in a representative subject. Weight of each 4 mm punch varied, ranging from 25.0-77.0 mg (mean 52.8±14.3 mg).


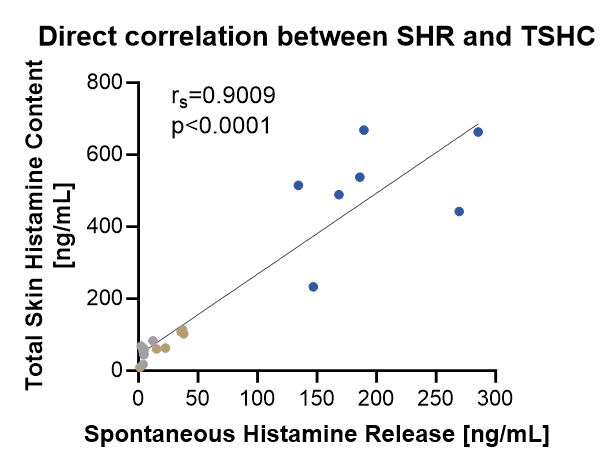


**Figure S2.** Spontaneous histamine release from 4 mm punches over 1 hour was directly correlated with total skin histamine content at physiologic temperature. Shown are the histamine data of individual punches from 3 donors (each represented by a different color).

**Figure S3.** Pertussis toxin (PTX) inhibited anti-IgE-mediated histamine release. Results are shown from paired 2.5 mm punches from a representative patient.

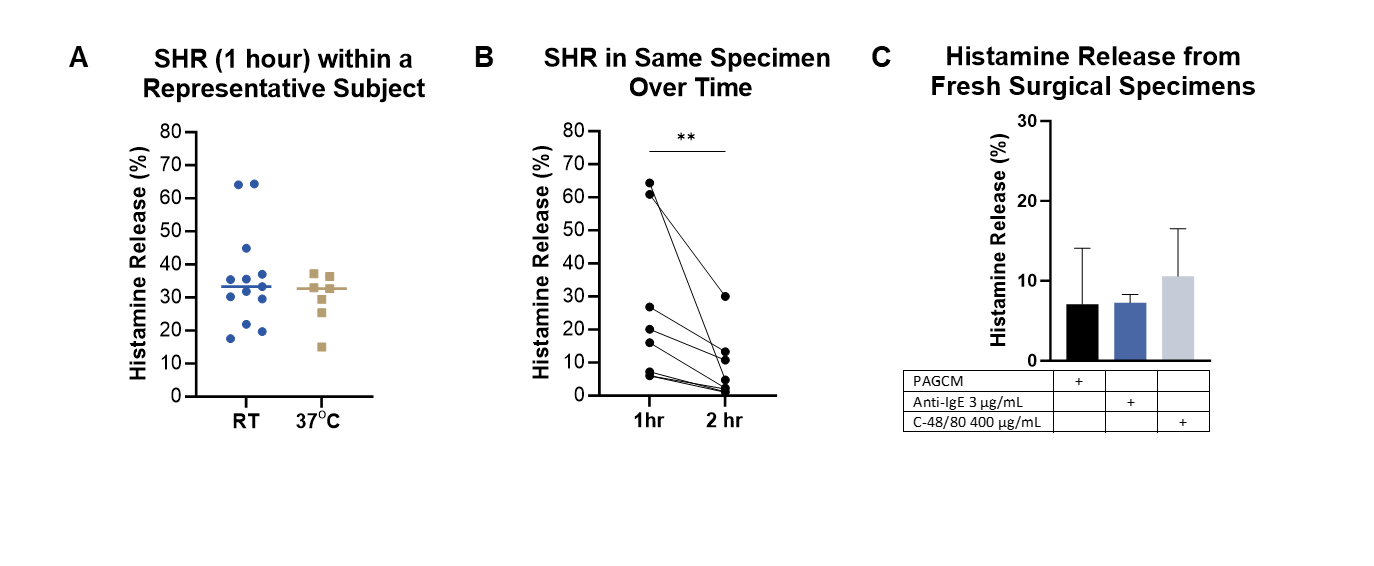


**Figure S4.** Spontaneous histamine release was markedly elevated in fresh surgical specimens. **(A)** Mean spontaneous histamine release from 4 mm punches was similar between specimens incubated at room temperature and those incubated at 37^o^C for 60 min. **(B)** SHR decreased by an average of 70.78±18.51% from the first hour to the second hour of incubation, yet it remained elevated at 8.00±9.93% during the second hour. Buffer was harvested and replaced at 60 min and 120 min. Data is shown for 5 subjects with each pair of connected dots representing one 4 mm punch biopsy. Wilcoxon paired sign rank test, p= 0.0078.  **(C)** Stimulated histamine release results (without EDTA washing) are shown for 2.5 mm biopsy pairs from 2 donors in duplicate.
